# Imaging Spatially Varying Dielectric Samples Using Tightly Coupled Dipole Array Based Near-Field Sensing

**DOI:** 10.3390/s26144607

**Published:** 2026-07-21

**Authors:** Thamer S. Almoneef

**Affiliations:** College of Engineering, Prince Sattam Bin Abdulaziz University, Al-Kharj 16273, Saudi Arabia; t.almoneef@psau.edu.sa

**Keywords:** metasurface sensor, electromagnetic coupling, microwave sensing, imaging, near-field characterization, dipole array

## Abstract

This paper presents a microwave sensing platform based on a 32-element dipole array designed for near-field dielectric contrast mapping. The sensor utilizes an 8×8 tightly coupled dipole array (TCDA) topology, where pairs of dipoles form unit cells that exploit electromagnetic coupling variations. A 32-way equal power divider network ensures uniform excitation across the aperture. Operating at 830 MHz, the dipole array exhibits high absorption (>90%), which enhances near-field intensity and sensitivity to surface perturbations. Experimental validation with dielectric samples, saline liquids of varying concentrations ($\epsilon_{r}\approx$ 70–78), and biological tissues demonstrates the array’s capability to map spatial variations in electromagnetic properties through rectified DC voltage shifts. When compared to a state-of-the-art multi-port Vector Network Analyzer (VNA) configurations, the proposed architecture offers a robust, low-complexity, proof-of-concept alternative by eliminating complex RF routing networks and multi-port switches.

## 1. Introduction

Microwave near-field sensing has emerged as a cornerstone for non-destructive testing, chemical analysis, and biological monitoring due to its non-ionizing nature and high sensitivity to dielectric variations [1,2,3]. The versatility of near-field microwave technology stems from its ability to exploit evanescent field components, which facilitate sub-wavelength spatial resolution and high sensitivity to local dielectric contrasts [4]. In the field of material characterization, near-field sensors are extensively utilized for the precise dielectric analysis of both solids and fluids. This is particularly evident in microfluidic applications, where the localization of electromagnetic energy within resonant structures allows for the detection of minute chemical variations in microliter sample volumes [5].

Beyond laboratory-scale material science, the non-ionizing and non-invasive nature of microwave radiation has catalyzed significant advancements in biomedical imaging. Researchers have successfully demonstrated the use of near-field probes for the differentiation of malignant and healthy biological tissues, leveraging the inherent permittivity differences caused by variations in water content and ionic concentration [6]. Notable applications include early-stage breast cancer detection, intracranial hemorrhage monitoring, and non-invasive glucose sensing [7]. Furthermore, in industrial environments, near-field microwave techniques are a cornerstone of non-destructive testing (NDT). They provide a robust mechanism for detecting subsurface defects, delamination in composite structures, and moisture ingress in civil infrastructure, all without necessitating physical contact with the Material Under Test (MUT) [8].

Recent advancements in metamaterials and metasurfaces have further pushed the limits of sensitivity by enabling sub-wavelength confinement of electromagnetic fields. Specifically, the utilization of high-quality-factor (*Q*) resonances has allowed for unprecedented precision in refractive index sensing [9,10]. However, while these single-element resonators excel at point-based detection, translating them into large-scale spatial imaging arrays remains a challenge due to the complexity of RF routing and the requirement for multi-port Vector Network Analyzer (VNA) measurements [11,12].

The requirement for spatial resolution over broad apertures has driven the adoption of mutual-coupling-based architectures and Tightly Coupled Dipole Arrays (TCDAs) [13,14,15]. Although typically reserved for wideband communication systems, the intense electromagnetic interaction between adjacent TCDA elements provides an advantageous platform for near-field detection [16,17,18]. When a sample interacts with the array, it disturbs the evanescent fringe fields between coupled dipoles; this interaction is captured through measurable shifts in the system’s mutual impedance or via localized mode-splitting behavior, enabling high-contrast dielectric contrast imaging [19].

A significant bottleneck in deploying these arrays for real-time spatial mapping applications is the data acquisition layer [20]. Traditional S-parameter characterization requires expensive and bulky equipment to monitor phase and magnitude shifts across multiple ports. To circumvent this, the integration of RF-to-DC rectification, often referred to as “rectennas,” at each sensing node allows for a simplified scalar readout [21,22,23,24,25]. By converting the coupled RF power directly into a DC voltage, the complex electromagnetic interaction is mapped onto a localized voltage shift, drastically reducing the backend hardware requirements [24,26].

To contextualize the exact contributions of this scalar architecture against traditional multi-port networks, a benchmarking matrix is presented in Table 1.

In this paper, we propose a 32-unit-cell TCDA operating at 830 MHz, specifically designed for near-field coupling-based proof-of-concept contrast mapping. The architecture utilizes an 8×8 grid where pairs of dipoles form unit cells that exploit sample-induced coupling perturbations. Unlike traditional reflection-based sensors, our design focuses on the variation of energy transfer between active and passive dipole pairs, which is subsequently converted to DC for localized spatial mapping. We demonstrate that the high absorption (>90%) at resonance significantly enhances the near-field intensity, making the system highly sensitive to spatial dielectric variations in liquids and biological tissues.

## 2. Theory of Tightly Coupled Dipole Arrays

A TCDA unit cell consists of an active dipole (port 1) and a passive dipole (port 2) separated by a sub-wavelength gap *G*. Their interaction is described by the two-port impedance matrix:

(1) $$\left[\begin{array}{c} V_{1}\\ V_{2} \end{array}\right]=\left[\begin{array}{cc} Z_{11} & Z_{12}\\ Z_{21} & Z_{22} \end{array}\right]\left[\begin{array}{c} I_{1}\\ I_{2} \end{array}\right],$$

where $Z_{12}=Z_{21}$ is the mutual impedance governing energy transfer between the two elements. The diagonal self-impedances $Z_{11}$ and $Z_{22}$ represent the isolated self-input impedances of each dipole element; due to the mechanically rigid structural microstrip frame, these terms remain highly stable under small localized environmental variations. When a material under test (MUT) of permittivity $\varepsilon_{MUT}$ is placed above the aperture, it perturbs the mutual impedance as follows [27]:

(2) $$\Delta Z_{12}\propto \int_{\mathrm{MUT}}\Delta \varepsilon \left(r\right)\;E_{1}\left(r\right)\cdot E_{2}\left(r\right)\;dV,$$

where $\Delta \varepsilon =\varepsilon_{MUT}-\varepsilon_{0}$ is the permittivity contrast, and $E_{1,2}$ are the unperturbed fields in the gap. This perturbation is strongest in the sub-wavelength gap region, where the evanescent fields of both dipoles overlap most intensely. The evanescent decay away from the aperture follows $e^{-\alpha z}$ with $\alpha =\sqrt{k_{t}^{2}-k_{0}^{2}}$, where $k_{t}$ is the transverse wave vector and $k_{0}$ represents the free-space wavenumber. This limits field penetration, confining the absolute reactive near-field region boundary up to approximately $z\approx \lambda_{0}/\left(2\pi \right)\approx 57.5\;\mathrm{mm}$ at 830 MHz.

Maximum sensing performance occurs at resonance ($f_{0}=830$ MHz), where the dipole array achieves >90% absorption:

(3) $$A\left(\omega \right)=1-{\left|S_{11}\right|}^{2}-{\left|S_{21}\right|}^{2},$$

where $S_{11}$ is the reflection coefficient, $S_{21}$ represents the inter-element transmission coupling coefficient, and ω is the operational angular frequency. At resonance, the input impedance is purely real, Im{Zin}|ω0=0. This condition maximizes near-field energy confinement in the gap, directly amplifying the sensitivity to Δε.

The perturbation $\Delta Z_{12}$ induced by the MUT is detected via RF-to-DC rectification at each passive dipole. The output DC voltage shift is:

(4) $$\Delta V_{DC}\approx \frac{\eta_{rect}\;R_{L}\;{\left|I_{1}\right|}^{2}}{\left(\right|Z_{22}\left|+\right|Z_{L}{\left|\right)}^{2}}\cdot |Z_{21}|\cdot |\Delta Z_{21}|,$$

where $\eta_{rect}$ is the conversion efficiency of the passive zero-bias Avago HSMS-286x Schottky rectifier diode network, and $R_{L}$ is the terminal DC video load resistance. Since $\Delta V_{DC}$ is linearly proportional to $|\Delta Z_{21}|$, and the coupling perturbation is spatially localized to each unit cell, the 32-pixel array maps the localized spatial dielectric contrast of the sample directly from scalar DC readouts.

## 3. Sensor Architecture and Operational Mechanism

**Figure 1:**
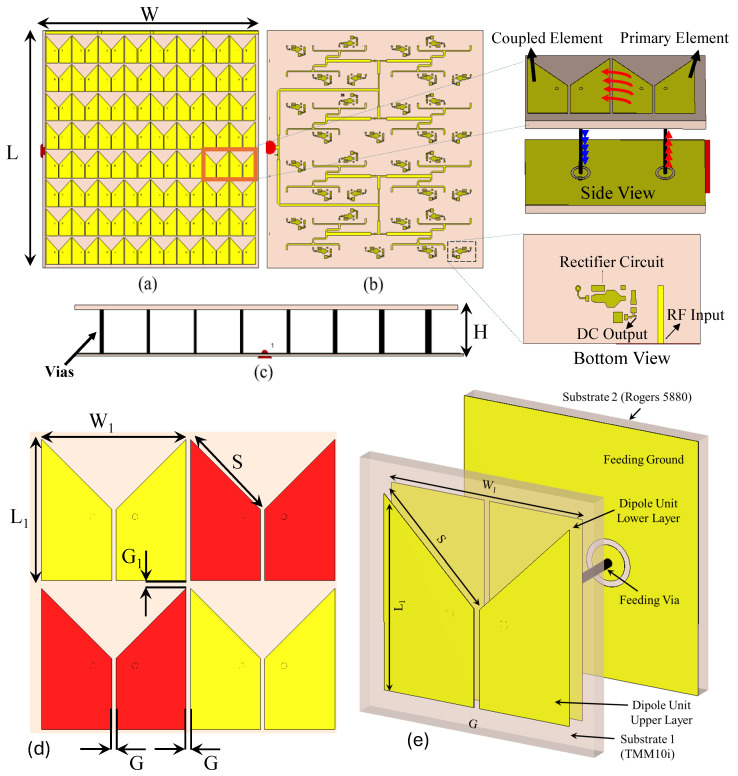
Proposed 32-pixel TCDA imaging array architecture: (**a**) front view of the 8×8 dipole grid forming the sensing aperture; (**b**) rear view showing the integrated 32-way power divider and rectification network; (**c**) cross-sectional schematic highlighting the multilayer PCB stack-up and vertical vias used for compact RF-to-DC signal routing. (**d**) a 2 × 2 sub array showing the inter-element spacing, G, between adjacent unit cells. (**e**) an isometric view of the single dipole element.

The architectural layout of the sensor array is illustrated in Figure 1a–c. The imaging system is realized through an 8×8 grid consisting of 64 dipole elements that are strategically partitioned into 32 independent unit cells. Each cell operates as a coupling-based transducer, pairing a primary active dipole with a secondary passive dipole. To ensure phase-coherent and uniform excitation across the entire aperture, the active dipoles are driven at a resonant frequency of 830 MHz via an integrated 32-way equal power divider (see inset of Figure 1). This multilayer Printed Circuit Board (PCB) configuration employs vertical vias to electromagnetically isolate the RF feed layer from the sensing interface, facilitating a high-density, compact integration of the distribution network and the transducers. The geometric configuration of the TCDA is defined by a 144mm×144mm overall footprint, utilizing a Rogers TMM 10i substrate with a thickness of 1.9mm. The unit cell architecture incorporates dipole elements with a length ($L_{1}$) of 16.6mm, a width ($W_{1}$) of 17mm, and a side length (*S*) of 11.67mm, all integrated with vertical vias at a height (*H*) of 18.48mm. Critical near-field coupling is maintained through precise inter-element gaps of G=0.5mm and $G_{1}=0.9\;\mathrm{mm}$.

The dipole array functions as a near-field spatial mapper that transduces the complex electromagnetic properties of a sample into a 4×8 matrix of DC signal levels. The fundamental sensing mechanism exploits sample-induced perturbations in the mutual coupling between adjacent dipoles. Because localized permittivity variations in the sample shift the inter-element evanescent fringe fields, these interactions are captured as scalar voltage fluctuations in the output. This modality allows the system to generate a direct “dielectric footprint” of the sample, providing proof-of-concept contrast mapping capabilities while bypassing the hardware complexity and computational overhead associated with traditional multi-port vector signal analysis.

The data acquisition (DAQ) framework for this 32-pixel system utilizes a distributed architecture to convert electromagnetic perturbations into a real-time digital stream. The sensing pixels are organized into four operational clusters, each containing eight cells (see inset of Figure 1). This parallel processing scheme interfaces each cluster with a dedicated STM32F4 microcontroller tasked with the continuous monitoring of rectified DC voltage shifts. As a sample disturbs the localized fringe fields, the resulting analog variations are captured by the microcontrollers’ onboard 12-bit Successive Approximation Register (SAR) analog-to-digital converters (ADCs). Subsequently, a central controller aggregates the digital data from all four processing nodes, synchronizing the 32-channel input into a unified data stream for spatial display.

## 4. Simulation Results and Discussion

**Figure 2:**
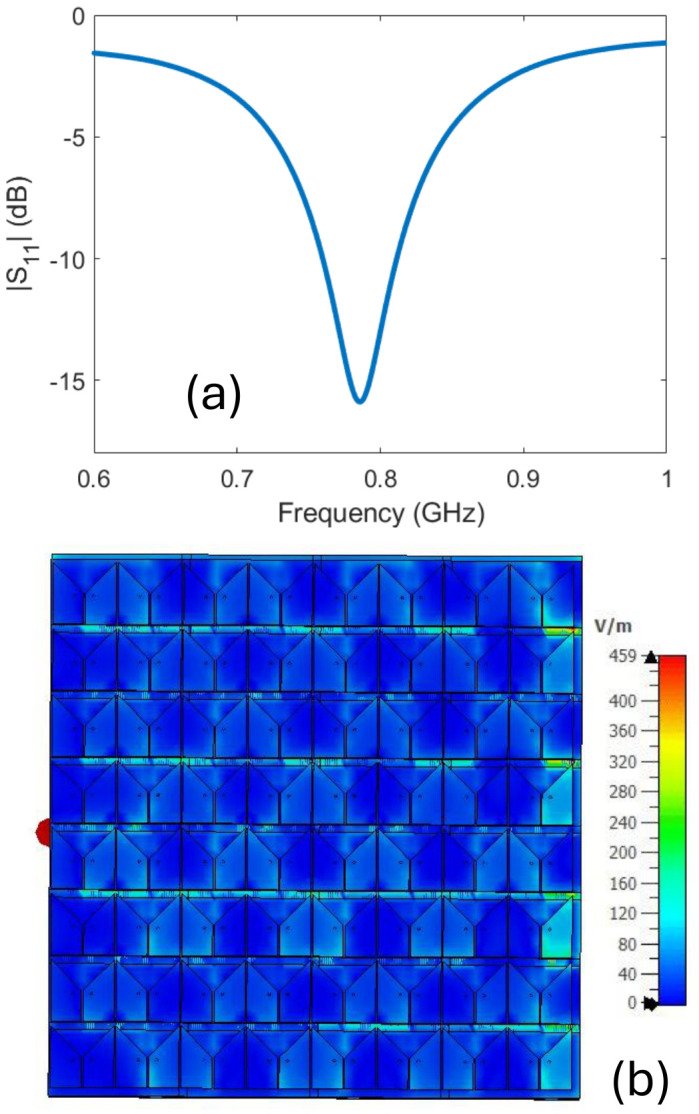
(**a**) Simulated response for the S-parameters due to unit cell and (**b**) electric field distribution on the antenna array at resonance frequency.

The electromagnetic properties of the constituent sensing pixels were evaluated via full-wave numerical simulations using the Finite Element Method (FEM) in Ansys HFSS, configured with an adaptive mesh convergence maximum delta S-parameter threshold error below −40dB. As illustrated in Figure 2a, the simulated reflection coefficient ($S_{11}$) reveals a distinct resonance at 830 MHz with a magnitude dipping to −16 dB. This profiles a robust impedance match between the multilayer feed network and the TCDA aperture, ensuring an effective input power coupling efficiency greater than 97.5%.

At this operational frequency, the unit cell demonstrates an absorption rate in excess of 90%, a critical characteristic that ensures incident RF energy is primarily confined within the reactive near-field of the dipole pair rather than being lost to reflection or radiation. This high degree of energy localization within the near-field is essential for performance sensing; it maximizes the interaction between the concentrated fringe fields and the material under test, thereby amplifying the sensor’s sensitivity to localized dielectric perturbations.

The high degree of energy localization within the TCDA is demonstrated by the simulated electric field distributions presented in Figure 2b. As illustrated, the field intensity reaches its peak concentration within the sub-wavelength coupling gap separating the active and passive dipoles. This spatial confinement is a direct consequence of the 830 MHz resonance, which serves to focus the electromagnetic energy into the reactive near-field.

These numerical results validate that the rectified DC output of each pixel acts as a probe for the localized dielectric environment. By maximizing the interaction between the concentrated fringe fields and the sample under test, the architecture provides a robust platform for proof-of-concept spatial contrast imaging, where minute permittivity-induced perturbations are efficiently transduced into measurable scalar voltage shifts.

To structurally maximize the spatial lateral resolution bounds in future revisions, the electrical pixel periodicity must be scaled down. This can be physically realized either by tightly reducing the sub-wavelength coupling gap boundaries (*G*) using high-density lithographic lines or by shifting the system to millimeter-wave bands to physically shrink overall unit-cell area footprints. Furthermore, spatial discrimination between an item’s absolute volumetric size and its constituent permittivity is achieved via gradient analysis: a small, high-permittivity object induces an abrupt single-pixel voltage deflection, whereas a larger, lower-permittivity sample projects a diffuse, multi-node signature.

## 5. Experimental Results and Discussion

**Figure 3:**
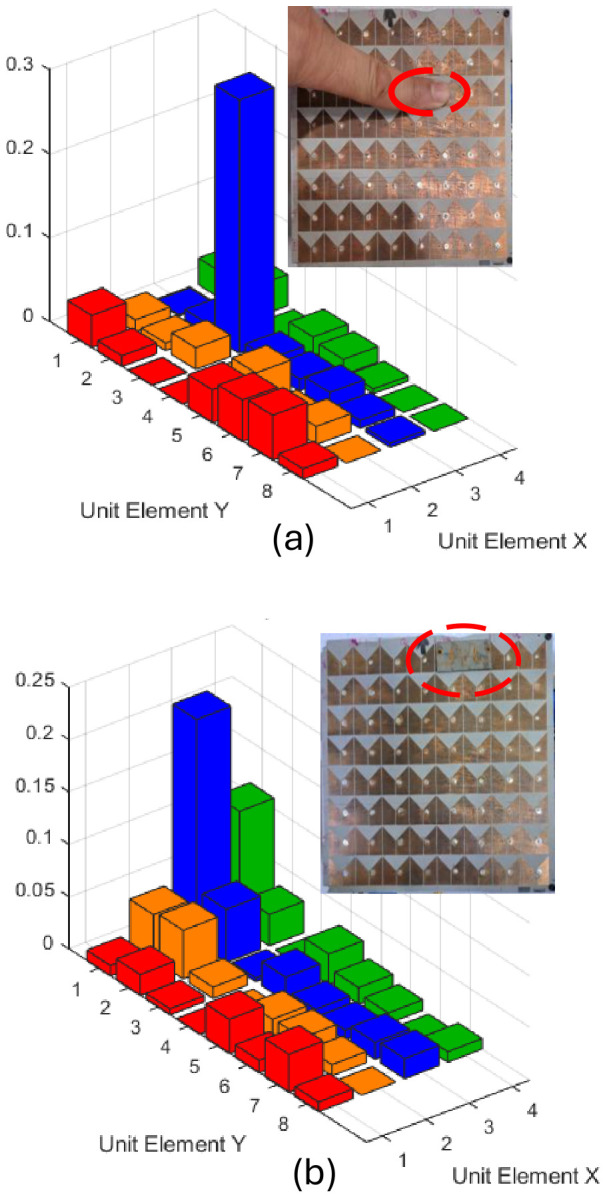
Near-field microwave imaging of a thin dielectric sample: (**a**) Finger placement on the dipole aperture to simulate a high-permittivity target and corresponding 8×4 spatial dielectric map. The localized intensity shift around unit element (3,3) demonstrates the system’s ability to resolve the spatial coordinates and dielectric contrast of the sample. (**b**) A dielectric strip placed on the 32-pixel dipole aperture and corresponding 8×4 spatial dielectric map generated from the rectified DC voltages.

To evaluate the proof-of-concept dielectric contrast mapping capabilities of the 32-pixel dipole array, several experimental trials were conducted using various objects under a continuous-wave source input power of +10dBm. The experimental setup, as described in Figure 3, was utilized to map the localized permittivity variations onto a spatial grid. Mutual isolation diagnostics confirm that the adjacent structural element cross-talk remains consistently below −18dB, preserving spatial selectivity floor bounds.

The sensitivity of the TCDA to high-permittivity biological samples was validated through a fingertip placement test. Human tissue, characterized by high water content and a high dielectric constant, serves as a significant perturbation to the evanescent fringe fields of the dipole pixels. As illustrated in Figure 3a, when a finger is placed on the dipole aperture, the inter-element coupling at the localized point of contact is significantly altered. The resulting spatial map in Figure 3a demonstrates a clear “hotspot” corresponding to the physical location of the finger around Row 3 and Column 3 of the unit element grid. The scalar voltage shift is localized to the specific unit elements (X,Y) directly beneath the sample, confirming the array’s spatial selectivity and low cross-talk between adjacent pixels.

To further evaluate the system’s capability for material contrast tracking and non-destructive testing, a thin dielectric strip was positioned across the dipole aperture. As shown in Figure 3b, the presence of the dielectric material significantly perturbs the inter-element coupling within the unit cells directly beneath it. The resulting 8×4 imaging grid clearly resolves the spatial profile of the strip. The imaging response indicates a high signal-to-noise ratio, with the peak intensity concentrated along the primary axis of the dielectric strip.

**Figure 4:**
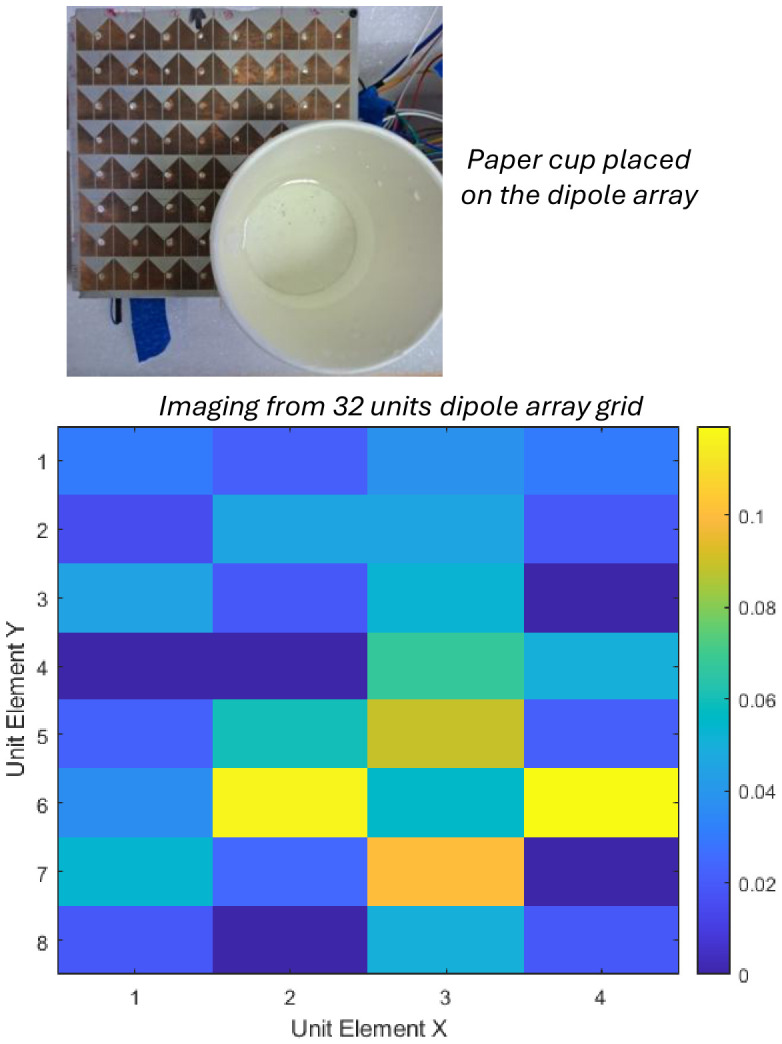
(**Top**) Photograph of a paper cup positioned on the TCDA sensing area. (**Bottom**) Resulting spatial dielectric map from the 32-pixel grid.

The sensor array’s ability to resolve objects with complex geometries and lower dielectric constants was further evaluated using a standard paper cup as the material under test (MUT). As illustrated in Figure 4, the presence of the cup in the dipole aperture induces a characteristic perturbation pattern in the near-field coupling of the underlying unit cells. Unlike the solid dielectric strip, the partially filled paper cup results in a more diffuse localized response.

The corresponding 8×4 spatial dielectric map successfully identifies the circular footprint and the contact region of the cup. The rectified DC voltage shifts are concentrated at the base of the cup, specifically appearing as a localized intensity variation in the central-lower region of the grid. In particular, the peak voltage shift recorded for the paper cup is approximately 0.1 V, which is significantly lower than the response observed for the high-permittivity human finger (0.3 V) or the dielectric strip (0.25 V). This quantitative difference demonstrates the system’s dynamic range and its capacity to distinguish between materials on the basis of their effective permittivity and the density of the sample interacting with the fringe fields.

## 6. Conclusions

In conclusion, a 32-cell microwave near-field sensor array based on coupling-perturbed dipole elements has been demonstrated at 830 MHz as a proof-of-concept contrast mapping engine. The system transforms complex near-field variations into readable scalar DC shifts, achieving a dynamic output voltage deflection up to 0.3V and maintaining adjacent inter-pixel isolation below −18dB without complex multi-port VNA hardware switches. While the current setup is limited by a fixed 17 mm physical pixel size and lacks absolute automated material classification loops, future investigations will focus on sub-wavelength grid downscaling, continuous calibration repeatability cycles, and active software drift-compensation models to bypass external noise.

## Figures and Tables

**Table 1:** Comparison of the proposed near-field sensor configuration with prior art array architectures.

Ref.	Frequency	Measurement Scheme	Hardware Complexity	RF Path Requirements
[11]	1–2 GHz	VNA/Multi-port S-parameters	High Matrix Complexity	Multi-port RF Switch Network
[20]	2.4 GHz	VNA/Multi-port Resonator	High Instrument Overhead	Complex Multi-port VNA Setup
This Work	830 MHz	Rectified Scalar DC Voltage	Low Onboard Footprint	Integrated Microcontroller ADCs

## Data Availability

The original contributions presented in this study are included in the article. Further inquiries can be directed to the corresponding author.
